# Supplementary material for: Epigenetic Modulation of Inflammatory Pathways in Myometrial Stem Cells and Risk of Uterine Fibroids
Source: Int J Mol Sci. 2023 Jul 19;24(14):11641. doi: 10.3390/ijms241411641 (PMC10380326; doi:10.3390/ijms241411641)
Supplement: Supplementary file 1 [file ijms-24-11641-s001.zip › ijms-2485213-supplementary.pptx]

## Slide 1
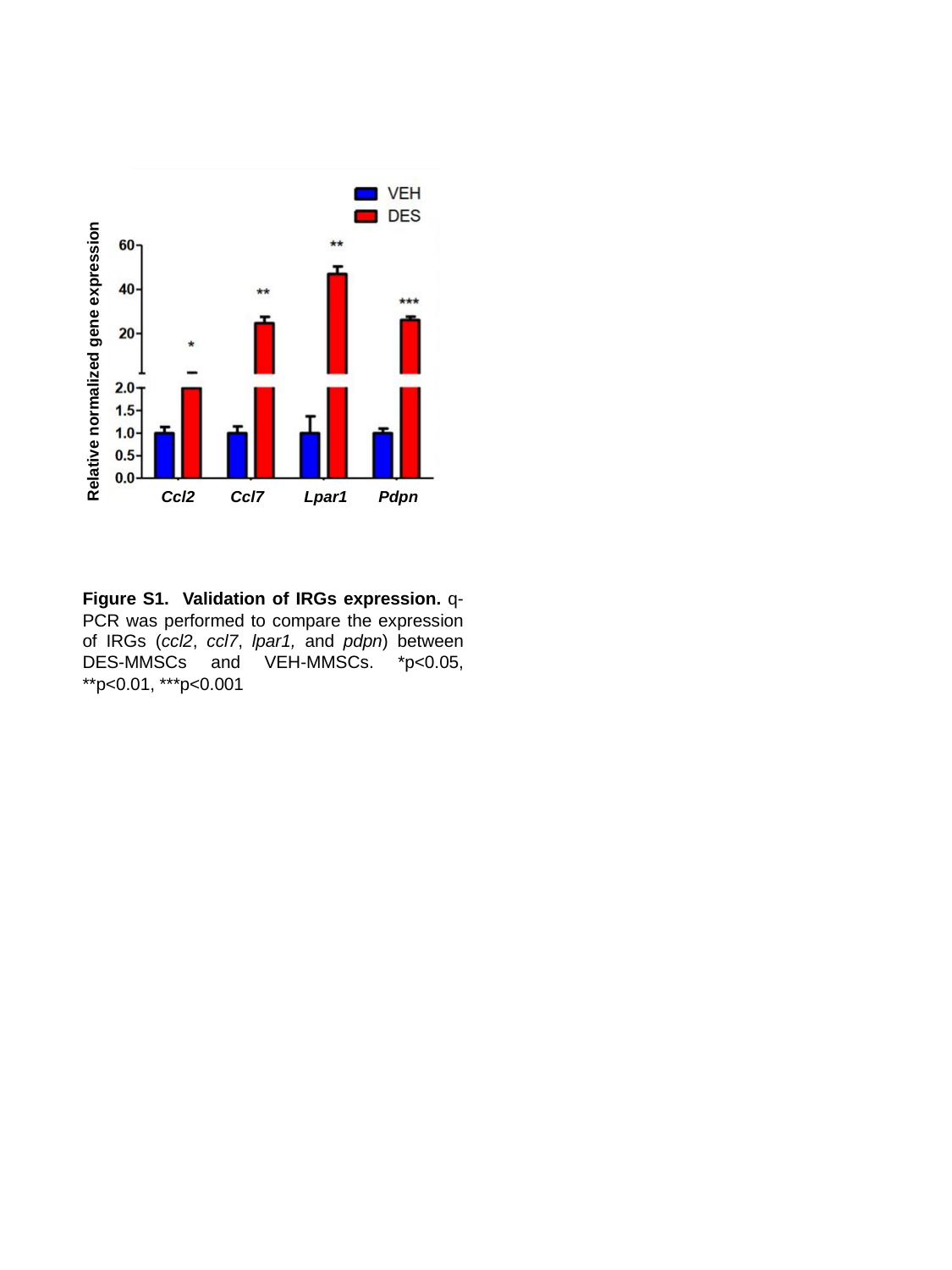

Figure S1. Validation of IRGs expression. q-PCR was performed to compare the expression of IRGs (ccl2, ccl7, lpar1, and pdpn) between DES-MMSCs and VEH-MMSCs. *p<0.05, **p<0.01, ***p<0.001
Relative normalized gene expression
Ccl2 Ccl7 Lpar1 Pdpn

## Slide 2
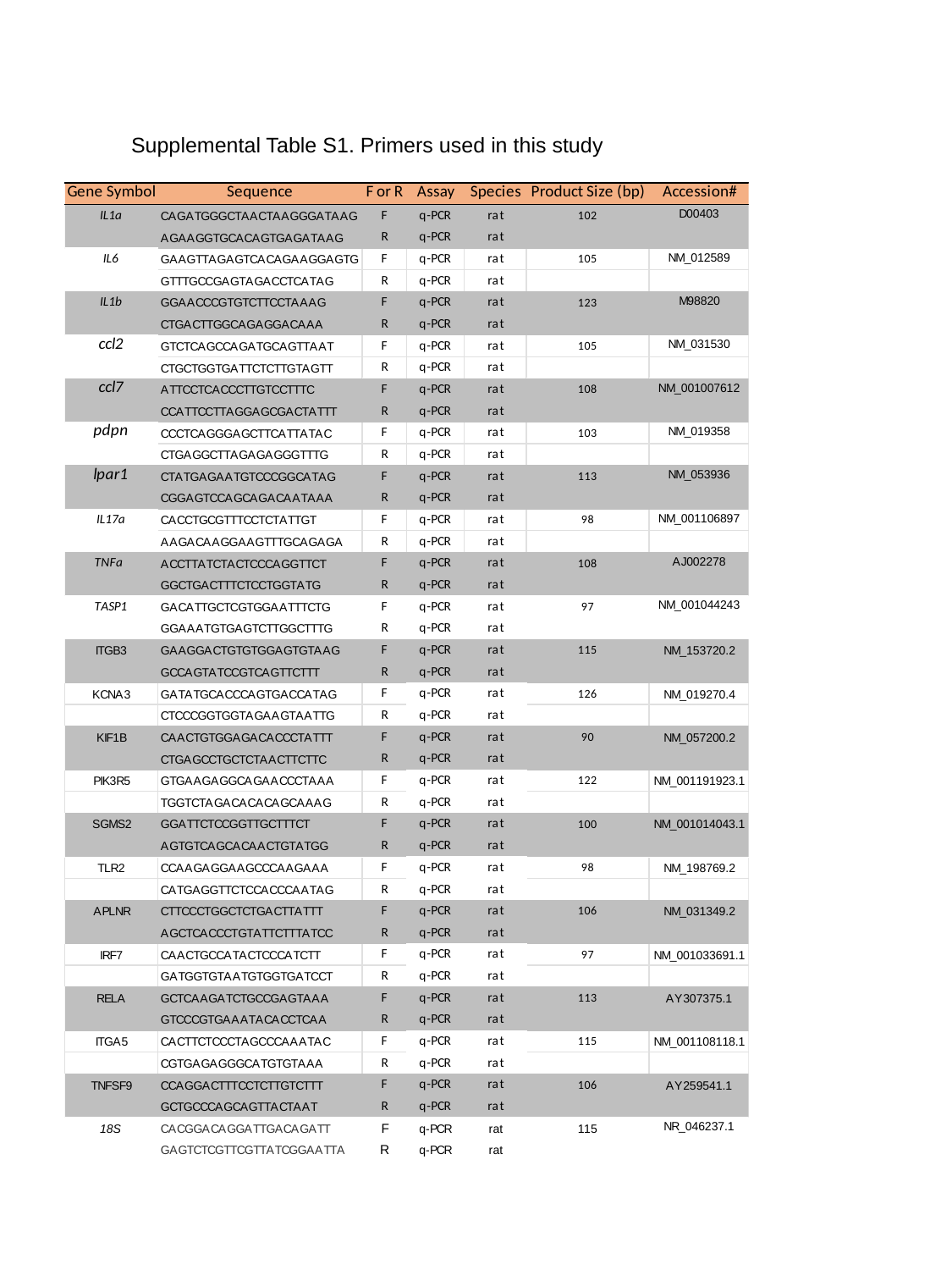

Supplemental Table S1. Primers used in this study
